# Anxiety unplugged: Effectiveness of an unguided, transdiagnostic, web-based intervention for anxiety disorders—A randomized controlled trial

**DOI:** 10.1016/j.invent.2025.100867

**Published:** 2025-08-08

**Authors:** Anna Baumeister, Lea Schuurmans, Steffen Moritz

**Affiliations:** a University Medical Center Hamburg-Eppendorf, Department of Psychiatry and Psychotherapy, Germany

**Keywords:** Internet-based intervention, Anxiety, Specific phobia, Self-help

## Abstract

**Background and aim:**

Anxiety disorders are the most common psychiatric condition, yet few patients receive adequate treatment, primarily due to high barriers to treatment (e.g., long waiting times, fear of stigmatization). Internet-based cognitive behavioral therapy (iCBT) programs have emerged as a promising approach to addressing this treatment gap, demonstrating effectiveness across various anxiety disorders. However, only a few studies have specifically addressed unguided iCBT. This trial investigated a six-week transdiagnostic unguided iCBT intervention designed to reduce anxiety and related symptoms.

**Methods:**

Randomized to an intervention group and a waitlist control group with care as usual, 318 participants were included in the analyses, which used ANCOVAs to assess changes in symptom severity of anxiety (primary) and related secondary outcomes (stress, depression and anxiety, quality of life, self-esteem).

**Results:**

Significant post-intervention reductions in anxiety, stress, and depression were found in the intervention group compared to controls at small to medium effect sizes (|Hedges's *g*| = 0.27–0.35). Concurrent ongoing psychotherapy negatively modified the treatment effect.

**Conclusion:**

The iCBT program proved effective overall in reducing anxiety and anxiety-related symptoms compared to care as usual. However, the program did not appear to have an add-on effect for individuals who were simultaneously receiving conventional psychotherapy. Further research is needed to investigate the effects in this subgroup in more detail.

## Highlights

• The iCBT intervention for anxiety was effective transdiagnostically, including for specific phobia.
• The effect was dependent on the current treatment of the participants (concurrent psychotherapy vs. no psychotherapy).
• None of the other analyzed moderators (e.g., treatment expectation) had an influence on the treatment effect.

## 1 Introduction

Anxiety disorders, particularly panic disorder with or without agoraphobia (PD-A/PD), social anxiety disorder (SAD), generalized anxiety disorder (GAD), and specific phobia (SP), are the most prevalent psychiatric disorder worldwide (Bandelow and Michaelis, 2015; Javaid et al., 2023). The persistent and excessive fear associated with anxiety disorders can result in significant impairment of functionality and quality of life (Wilmer et al., 2021) as well as low self-esteem, especially in SAD (Iancu et al., 2015).

Despite the high burden of anxiety disorders with respect to daily life impairment, affected individuals rarely receive adequate treatment, particularly cognitive behavioral therapy, which is the gold standard in treating anxiety disorders. A worldwide evaluation of the treatment gap for anxiety disorders showed that only one fourth of affected individuals receive any kind of treatment and only 10 % receive adequate (i.e., pharmacotherapy or psychotherapy) treatment (Alonso et al., 2018). The reasons for this substantial treatment gap are low help-seeking behavior and major treatment barriers, such as desire for autonomy, unavailability of treatment (e.g., long waiting times), and fear of stigmatization (Heinig et al., 2021).

Online programs that are mostly based on cognitive behavioral therapy (iCBT programs) have been developed to address this treatment gap. Internet-based interventions, especially when designed to be unguided (i.e. without professional guidance), have advantages over conventional face-to-face therapy as they are easily available and offer privacy and autonomy (Laux, 2017). A meta-analysis conducted by Pauley et al. (2023) examined randomized controlled trials (RCTs) on internet-based interventions for anxiety disorders over two decades, assessing 47 trials with almost 5000 participants. They conducted extensive analyses that included pooled effect sizes, subgroup analysis for disorder (PD/PD-A, SAD, GAD), guidance (guided/unguided), and recruitment (clinical or community recruitment setting), showing positive results overall (treatment vs. inactive controls, *g* = 0.80) and without differences between subgroups, with effect sizes ranging between *g* = 0.61 and 1.37. Notably, interventions with or without guidance did not differ in terms of effectiveness (Pauley et al., 2023). This extensive meta-analysis shows the promise of this kind of intervention for anxiety disorders and other highly prevalent disorders with large treatment gaps. However, only two of the 47 trials included in the meta-analysis evaluated transdiagnostic unguided iCBT programs.

One of these studies, by Bell et al. (2012), was conducted in a naturalistic setting and included patients with GAD, PD, and SAD in secondary care. The program was beneficial regarding work and social functioning as well as other anxiety-related outcomes (e.g., worry, social anxiety), with moderate effect sizes compared to waitlist controls. Although described as an unguided program set in real-world conditions, participants in both groups had contact with non-clinical study staff every two weeks during the intervention period of 12 weeks (Bell et al., 2012). Another study conducted by Berger et al. (2017) investigated a transdiagnostic web-based iCBT program for SAD, GAD, and PD-A/PD. Again, the program was effective in reducing anxiety and anxiety-related symptoms compared to care as usual. Notably, Pauley et al. (2023) did not include any studies on SP as the primary diagnosis in their meta-analysis, indicating that evidence for internet-based interventions as a treatment approach for SP is still scarce. A preliminary meta-analysis conducted by Mor et al. (2021) found nine studies on this topic, of which five were RCTs with adults. Even though effects of internet-based interventions for SP seem promising, the authors concluded that due to the relatively small number and somewhat poor quality of the studies, more research is needed.

The current trial evaluated a transdiagnostic, unguided web-based iCBT intervention. An earlier version of the intervention has already been evaluated in an RCT with 179 participants with anxiety disorder (agoraphobia [AP]/PD, PD, SAD, SP; Schröder et al., 2017). The intervention group showed significant improvement in symptom severity compared to waitlist controls (η_p_^2^ = 0.03–0.05, *p* < .05). In the study by Schröder et al. (2017), the treatment effect was moderated by attitude toward online interventions, with a more positive attitude leading to greater symptom reduction. As evidence of factors influencing the efficacy of iCBT is still scarce, we decided to include further potential moderators in addition to attitude toward online interventions. Treatment expectation has been shown to have predictive value for the treatment response in CBT for individuals with public speaking anxiety (Price and Anderson, 2012). Further, as comorbid disorders are highly prevalent in patients with anxiety disorders (Koyuncu et al., 2019; Magee et al., 1996), comorbidity is considered in this study to be a plausible exploratory moderator as higher comorbidity may increase the symptom burden and potentially influence how individuals respond to the intervention.

The six-week program evaluated in the present study aims to reduce anxiety symptoms as well as depression and stress, increase self-esteem, and improve quality of life in patients with PD/PD-A, SAD, and—particularly noteworthy—SP over six weeks. Moderation and subgroup analyses in this trial considered various potential influencing factors.

## 2 Methods

The two-arm randomized controlled trial consisted of three assessments for both the intervention group (IG) and the waitlist control group (WCG).

The post assessment (T1) took place six weeks after the baseline assessment (T0), followed by a follow-up assessment (T2) six weeks after T1 (12 weeks after T0). The study was carried out as an online study using the survey software Qualtrics®. The sample size calculation using G*Power yielded an optimal sample size of 310 participants, based on an assumed effect of *d* = 0.40, corrected for a potential dropout rate of 20 % (d*(1–0.2) = 0.32), an alpha of 5 %, and a power of 80 %.

The study was approved by the Local Psychological Ethics Committee of the University Medical Centre Hamburg-Eppendorf (Germany, ID: LPEK-0606). The study was pre-registered in the German Clinical Trials Register (www.drks.de; DRKS00031668).

### 2.1 Procedure

Recruitment was carried out through online social forums for anxiety patients, social media channels, and in collaboration with medical care centers in Germany. Eligible participants (a) had one of the following ICD-10 diagnoses: F40.0× (AP with/without PD), F40.1 (SAD), F40.2 (SP) or F41.0 (PD), (b) were between 18 and 75 years old, (c) gave informed consent to participate in the study, (d) had access to the internet, (e) had sufficient knowledge of the German language, and (f) had not had any changes in treatment for at least four weeks prior to randomization and planned to maintain this over the study period if possible. Individuals were excluded from the study if they had (a) a diagnosis of a psychotic or bipolar disorder, (b) acute suicidal behavior within the last four weeks, (c) dementia, (d) alcohol or substance dependence within the last six months, or (e) were currently taking high-potency and/or atypical antipsychotics. Interested adults with a diagnosis matching the inclusion criteria received information about the study and an online informed consent form. After consenting to participate, they were directed to the baseline survey. Individuals who did not consent and participants who were screened positively for one of the exclusion criteria were automatically excluded from the survey. Upon successful completion of the baseline survey, the participants were automatically randomized into one of the two groups (IG, WCG). Randomization was stratified based on the participant's current care situation (with or without ongoing psychotherapy), with each category constituting a proportionate 50 % of the total sample (randomization ratio 1:1). This approach ensured equal randomization for both subgroups.

Participants in the WCG were provided with access to the intervention after completion of the study period. Throughout the course of the study, participants in both groups received care as usual (CAU). CAU included ongoing engagement in any regular therapies or treatments (i.e., medication) per their usual care and clinical needs, if any, with no restrictions imposed due to study participation.

### 2.2 Intervention

The Novego® program for anxiety disorders is based on established methods of cognitive behavioral therapy as well as approaches from systemic therapy and mindfulness-based therapy. It consists of six weekly modules, each with a duration of approximately 45 to 60 min. The modules include psychoeducational texts, audio files, videos, exercises, and illustrations, as well as diary templates. Users have the option of receiving automated, motivational email and text message reminders to use the program. This improves adherence to the program, but it remains an unguided intervention. The program is made available to participants for a period of at least one year following the initial six-week intervention period, with work materials, documents, and exercises available for download to ensure long-term accessibility. The content and images in the transdiagnostic program are automatically compiled and tailored to the participant's personal situation based on an initial questionnaire that assesses symptoms and sociodemographic factors. Participants select in the initial questionnaire the symptom description that best reflects their personal experience. The available options include brief but intense anxiety attacks accompanied by somatic symptoms such as heart palpitations, trembling, and loss of control; persistent worry associated with catastrophic thinking and inner restlessness; or pronounced social anxiety and avoidance of interpersonal interactions. Based on this selection, participants are assigned to one “focus area” (e.g., panic and phobias or social anxiety). All focus areas are grounded in the same cognitive-behavioral principles and interventions, and they are supplemented by content specifically addressing the characteristics of the selected anxiety type. The program consists of six sequential modules. The first module introduces the program structure and provides psychoeducation on various anxiety disorders, along with initial guidance on exposure-based exercises. The second module addresses the destigmatization of anxiety, introduces structured problem-solving training, and includes a guided reflection exercise. The third module focuses on cognitive restructuring of dysfunctional thoughts, acceptance of anxiety, and relaxation techniques. The fourth module targets the reduction of safety behaviors, incorporates guided imagery of an anxiety-free life, and introduces mindfulness-based strategies. The fifth module promotes engagement in positive activities, encourages a humorous approach to anxiety, and includes training in social skills. The final module offers a reflection on the previous weeks, deepens problem-solving strategies, and supports the development of individualized relapse prevention plans.

### 2.3 Measures

#### 2.3.1 Primary outcome

##### 2.3.1.1 Beck Anxiety Inventory (BAI)

The change in the severity of anxiety symptoms from T0 to T1 was defined as the primary endpoint assessed by the BAI (Beck et al., 1988). The BAI is a general self-report questionnaire that measures the severity of anxiety symptoms within the last week with 21 items on a four-point Likert scale (0 to 3). The total score ranges from 0 to 63, with higher scores indicating greater severity of anxiety symptoms. The internal consistency of the BAI in this study was satisfactory (α = 0.89).

#### 2.3.2 Secondary outcomes

##### 2.3.2.1 Depression Anxiety Stress Scale – 21 (DASS-21)

The DASS-21 (Lovibond and Lovibond, 1995) is a multidimensional self-assessment questionnaire developed for the simultaneous assessment of depression, anxiety and stress. It consists of 21 items, with each of the three scales comprising seven items that are rated on a four-point Likert scale (0 to 3). The total score ranges from 0 to 63, with higher scores indicating more severe symptoms. The scale demonstrated excellent internal consistency, with Cronbach's α = 0.91.

##### 2.3.2.2 Rosenberg Self-Esteem Scale (RSES)

The RSES (Rosenberg, 1965) is a 10-item self-report questionnaire that measures self-esteem. Participants indicate their level of agreement or disagreement with each item on a four-point Likert scale ranging from (1) “strongly agree” to (4) “strongly disagree.” The total score is then calculated; values range from 10 to 40, with higher scores indicating greater self-esteem. The internal consistency of the scale (Cronbach's α = 0.90) was good.

##### 2.3.2.3 World Health Organization Quality of Life – BREF (WHOQOL-BREF)

The WHOQOL-BREF (Whoqol Group, 1998) is a short version of the World Health Organization Quality of Life – 100 (WHOQOL-100) questionnaire. It measures the subjective quality of life in self-report on four subscales (psychological well-being, physical well-being, social relationships, and environment). The items are scored on a five-point Likert scale (1 to 5). The calculation of an overall score on a scale between 0 and 100 is performed by conversion of the subscales' mean values—(subscale mean minus 4) x (100/16)—with higher values indicating a better quality of life. The internal consistency was good (Cronbach's α for the overall score = 0.90).

##### 2.3.2.4 Liebowitz Social Anxiety Scale – Self Report (LSAS-SR)

The LSAS-SR (Liebowitz, 1987) is a self-report questionnaire used to assess the severity of social phobia. The questionnaire contains 24 items that are answered on a four-point Likert scale (0 to 3), with higher scores indicating more severe social phobia. The questionnaire demonstrated good psychometric properties overall (Cronbach's α = 0.97).

##### 2.3.2.5 Attitude toward Online Interventions (APOI)

Participant's attitude toward internet-based interventions at baseline was assessed using the APOI (Schröder et al., 2015). The APOI consists of four subscales (“skepticism and risk perception”, “confidence in effectiveness,” “technologization threat,” and “anonymity benefits”). Items are rated on a 5-point Likert scale, with higher scores indicating a more positive attitude. The internal consistency was low in this study (Cronbachs' α = 0.51).

##### 2.3.2.6 Treatment Expectation Questionnaire (TEX-Q)

Treatment expectations at baseline were assessed using the TEX-Q (Alberts et al., 2020), which consists of 15 items assessing expectations of a treatment. The items are rated on an 11-point Likert scale (0 to 10). After inverting negative items, an overall mean value can be calculated, with higher values representing a more positive treatment expectation. The internal consistency was good (Cronbach's α = 0.82).

##### 2.3.2.7 Web Screening Questionnaire (WSQ)

Comorbid disorders were assessed using the WSQ (Donker et al., 2009). The WSQ screens with 15 self-report items common mental disorders (depression, generalized anxiety disorders, PD-A/PD, AP, SP, posttraumatic stress disorder, obsessive compulsive disorder, alcohol abuse/dependence, and suicidal ideations).

##### 2.3.2.8 Client Satisfaction Questionnaire (CSQ-8)

Satisfaction with the intervention was assessed using the CSQ-8 (Schmidt and Wittmann, 2002). The items are rated on a 4-point Likert scale, and a total score ranging from 8 to 32 can be calculated. After recoding negative items, a higher score represents higher satisfaction. The internal consistency in this study was good (Cronbach's α = 0.94).

### 2.4 Statistical analyses

Statistical analyses were conducted using IBM SPSS® version 29 and R version 4.1.1. Analyses of the primary and secondary endpoints were performed using analyses of covariance (ANCOVA). The difference values between baseline and post survey (six weeks after baseline) served as dependent variables. The baseline values were included in the analysis as covariates to control for regression to the mean. The effect sizes of the analyses were calculated using Hedges's *g*. In addition, least square mean differences (LS MD; including the 95 % confidence interval, CI) are reported. LS MD are particularly suitable when differences between groups are to be calculated in the context of a model-specific approach, where controlling for covariates is important, which is the case in this study. They provide a transparent effect size related to the original scale, which is useful in clinical trials in addition to standardized effect sizes like Hedges's *g*.

Intention-to-treat analyses (ITT) represented the primary analyses. Missing values were imputed using the reference-based jump-to-reference (J2R) method (Carpenter et al., 2013) with *m* = 150 imputation sets, with age, gender, care situation (current psychotherapy yes/no), and educational level serving as covariates. Under the J2R assumption, participants in the intervention group with missing data after the intervention are assumed to have future outcomes that follow the course of the reference group (control group) from the time of discontinuation. This conservative strategy corresponds to plausible clinical scenarios in which participants discontinue the intervention and subsequently receive standard treatment (e.g., CAU). The J2R method reduces the risk of overestimating treatment effects due to missing data as it avoids optimistically biased assumptions about sustained benefits after treatment discontinuation. This approach was specified in our pre-registration (see www.drks.de; DRKS00031668) and is recommended in current methodological guidelines to improve the robustness and transparency of trial results (Carpenter et al., 2013; Cro et al., 2020; White and Carpenter, 2023). To evaluate the robustness of the results, the effectiveness analyses (ANCOVAs) were additionally conducted with multiple imputation by chained equations (MICE), based on the assumption that data were missing at random, with *m* = 50 imputations sets using age, gender, educational level, relationship status, care situation, and respective baseline scores as predictors. Estimates for both imputation strategies (J2R and MICE) were pooled by Rubin's Rule.

Further sensitivity analyses were conducted with the complete cases (CC; data from participants with complete data sets) and the per protocol population (PP). PP was defined as data from participants with complete data sets and from participants in the IG who logged in to the program at least four times. Requiring a minimum of four logins over a six-week period is a realistic and flexible engagement criterion, one that is likely to be achievable even for participants with professional, family, or other obligations. Given the modular structure of the intervention (i.e., one module per week over six weeks), four logins may indicate that participants were exposed to at least two-thirds of the program content, thereby ensuring a minimum level of engagement necessary for evaluating potential effects. The Reliable Change Index (RCI) was calculated in the CC sample to assess clinical relevance.

In addition to the complete sample, exploratory subgroup analyses were conducted with the ITT sample for gender (male, female), diagnosis (AP, PD, SAD, SP), care context (with psychotherapy, without psychotherapy) and care as usual (with medication, without medication). Furthermore, moderation analyses to identify variables that might influence the change in anxiety symptoms over time were conducted. Potential moderator variables were attitude toward online interventions, treatment expectations, and the presence of comorbid disorders. The moderation analyses were calculated with the PP data using the SPSS® macro PROCESS (developed by A. Hayes).

## 3 Results

**Fig. 1:**
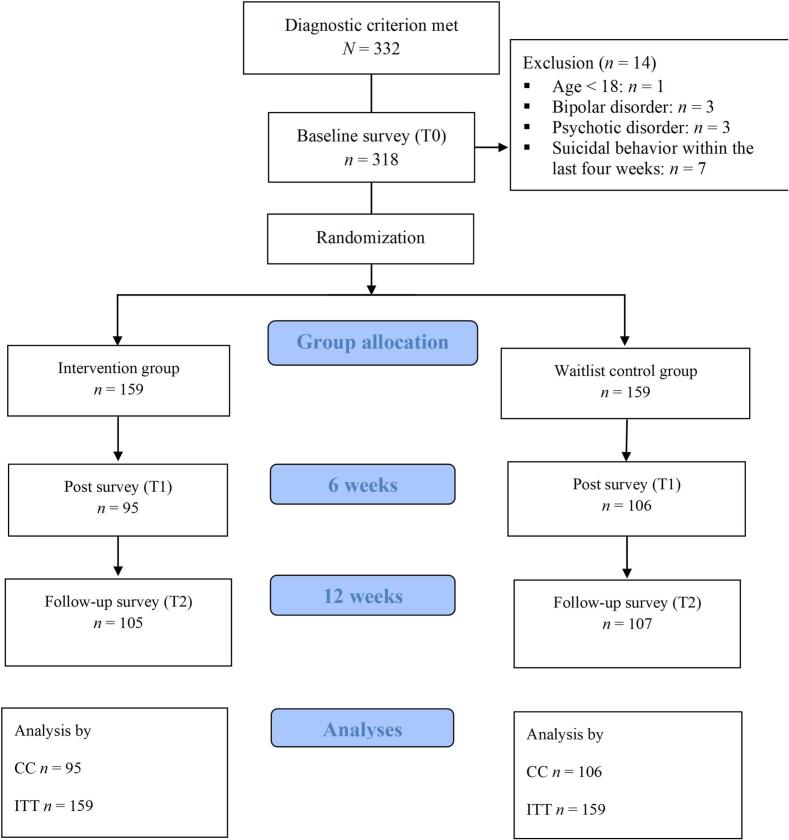
Flow chart.

A total of 332 people participated in the baseline survey, of whom 318 were randomized. At T0, 14 participants were excluded from the study (see Fig. 1).The participants who were included in the study were randomly assigned to either the IG (*n* = 159) or the WCG (*n* = 159). The sociodemographic and psychopathological characteristics of the sample can be found in Table 1.

**Table 1:** Sample demographics, with baseline comparisons between the groups.

	Total (*N* = 318)	WCG (*n* = 159)	IG (*n* = 159)
*Demographic characteristics, n (%)*			
Gender			
Female	253 (71.9 %)	129 (81.1 %)	124 (78.0 %)
Male	63 (26.7 %)	30 (18.9 %)	33 (20.8 %)
Diverse	2 (1.3 %)	0 (0 %)	2 (1.2 %)
Age in years, *M (SD)*	41.8 (12.8)	43.0 (13.8)	40.5 (11.7)
Higher education	196 (61.6 %)	91 (57.2 %)	105 (66.0 %)
*Psychopathology, M (SD)*			
Anxiety (BAI)	30.6 (2.0)	30.7 (10.4)	30.5 (11.5)
Social anxiety (LSAS)	63.1 (33.7)	64.8 (35.2)	61.5 (32.2)
Depression, anxiety, stress (DASS-21)	33.4 (12.1)	33.2 (12.0)	33.6 (12.3)
Self-esteem (RSES)	15.5 (6.2)	15.5 (6.2)	15.4 (6.2)
Quality of life (WHOQOL-BREF)	39.2 (1.9)	39.3 (19.5)	39.1 (20.2)
*Diagnosis, n (%)*			
Agoraphobia (F40.00)	16 (5.0 %)	9 (5.7 %)	7 (4.4 %)
Agoraphobia with panic disorder (F40.01)	95 (29.9 %)	40 (25.2 %)	55 (34.6 %)
Panic disorder (F41.0)	126 (39.6 %)	68 (42.8 %)	58 (36.5 %)
Social anxiety disorder (F40.1)	56 (17.6 %)	30 (18.9 %)	26 (16.4 %)
Specific phobia (F40.2)	25 (7.9 %)	12 (7.5 %)	13 (8.2 %)
*Psychotherapy, n (%)*			
With psychotherapy	132 (41.5 %)	66 (41.5 %)	66 (41.5 %)
No psychotherapy	186 (58.5 %)	93 (58.5 %)	93 (58.5 %)
*Psychopharmaco therapy. n (%)*			
No intake	189 (59.4 %)	97 (61.0 %)	92 (57.9 %)
Regular intake	129 (40.6 %)	62 (39.0 %)	67 (42.1 %)
*Other, M (SD)*			
Comorbid diagnoses (WSQ)	3.1 (1.2)	3.2 (1.3)	3.0 (1.1)
Attitude toward online interventions (APOI)	55.6 (5.3)	55.6 (5.0)	55.5 (5.5)
Treatment expectation (TEX-Q)	6.7 (1.2)	6.7 (1.2)	6.7 (1.1)

*Note*. APOI = Attitude toward psychological online interventions; BAI = Beck Anxiety Inventory; DASS-21 = Depression Anxiety Stress Scale; IG = intervention group; LSAS = Liebowitz Social Anxiety Scale; RSES = Rosenberg Self-Esteem Scale; TEX-Q = Treatment Expectation Questionnaire; WCG = waitlist control group; WHOQOL-BREF = World Health Organization Quality of Life Brief Questionnaire; WSQ = Web Screening Questionnaire.

### 3.1 Sample

The sample was predominantly female (71.9 %), had an average age of 41.8 years, and had a high level of education, with 61.6 % of participants having at least a university entrance qualification (see Table 1). Almost 50 % of the participants were not undergoing psychotherapeutic treatment at the time of the baseline survey. Slightly more than 40 % of the sample (40.6 %) regularly took psychotropic medication (see Table 1).

A total of 205 participants (64.5 %) completed the post survey (T1, six weeks after baseline), and 212 (66.7 %) completed the follow-up survey (T2, 12 weeks after baseline). Surveys were also included in the analyses if individual questionnaires were available in full (individual items were not imputed), resulting in a slightly increased retention rate of 67.3 % for the primary endpoint (BAI at T1).The retention rate at the post survey did not differ significantly between the IG and the WCG (χ^2^(1) = 0.87, *p* = .35). Additionally, there were no statistically significant differences observed in the current care situation (χ^2^(1) = 0.00, *p* = .97), educational status (χ^2^(4) = 2.49, *p* = .65), or anxiety symptom severity at baseline (BAI; *t*(316) = 0.69, *p* = .49).

### 3.2 Intervention adherence and satisfaction

The IG participants logged in to the program on average 11 times (*M* = 11.4, *SD* = 11.02; range: 0–55) over the intervention period of six weeks. A total of 26 participants (16.4 %) did not log in to the program even once. On average, three of the six modules were completed within the intervention period of six weeks (*M* = 2.9, *SD* = 2.12).

The CSQ-8 (range 8–32) was used to assess satisfaction with the program at T1, which averaged 22.9 (*SD* = 4.94). Among the IG participants, 72.9 % indicated that they would use the program again if they needed support, and 78.1 % indicated they would recommend the program to others.

### 3.3 Effectiveness

#### 3.3.1 Intention to treat

The ANCOVA showed a significantly greater reduction for the primary endpoint (change in the BAI total score at time T1) in the IG compared to the WCG (*p* = .023). Additionally, a significantly greater reduction in the secondary endpoints at T1 for social anxiety symptoms (LSAS, *p* = .003) and depression, anxiety, and stress symptoms (DASS-21, *p* = .010) was observed in the IG compared to the WCG. The results are displayed in Table 2. The effects were sustained for the BAI and the LSAS at follow-up (see Supplements, Table A). Further, for self-esteem (RSES), a significantly greater increase was found in the IG compared to the WCG at follow-up (see Supplements, Table A), but not at T1 (*p* = .082; see Table 2).

**Table 2:** Differences between the groups of the intention-to-treat sample at T1 (ANCOVAs with baseline values and care context as covariates), imputed with jump-to-reference, showing least square mean differences (LS MD) with 95 % CI, *p*-value and effect size η_p_^2^ and Hedges's *g*.

		95 %CI			*p*	η_p_^2^	Hedges's *g*
	LS MD	Lower	Upper	Statistics			
**BAI**	2.64	0.37	4.91	*F*(1;2107.14) = 5.2	0.023	0.023	−0.27
**LSAS**	6.85	2.33	11.37	*F*(1;3278.50) = 8.83	0.003	0.035	−0.35
**DASS-21**	3.22	0.78	5.66	*F*(1;2804.52) = 6.73	0.010	0.028	−0.31
**RSES**	−0.79	−1.69	0.10	*F*(1;2198.78) = 3.03	0.082	0.014	0.18
**WHOQOL**	−3.11	−7.27	1.06	*F*(1;3611.86) = 2.16	0.142	0.009	0.22

*Note*: BAI: Beck's Anxiety Inventory; LSAS: Liebowitz Social Anxiety Scale; DASS-21: Depression Anxiety Stress Scale; RSES: Rosenberg Self-Esteem Scale; WHOQOL: World Health Organization Quality of Life Brief Questionnaire.

The WHOQOL-BREF at T1 and T2 showed no significant improvement in the IG compared to the WCG.

#### 3.3.2 Sensitivity analyses

The MI-based ITT analyses also confirmed the effects of the primary analyses, with larger effect sizes except for the WHOQOL (see Supplements, Table B). Regarding the CC sample analyses, the sensitivity analyses supported the results of the primary ITT analyses (see Supplements, Table C). In the PP population (*n* = 85 IG, *n* = 106 WCG), the results of the previous analyses were confirmed, with overall larger effect sizes (see Table 3). The results remained consistent in the follow-up (see Supplements, Tables D-F).

**Table 3:** Differences between the groups of the per protocol sample over time (baseline T0 to post T1), with mean values and standard deviations (*N* = 191).

	IG (*n* = 85)		WCG (*n* = 106)		Differences between the groups from baseline to post		
	Baseline *M* (SD)	Post *M* (SD)	Baseline *M* (SD)	Post *M* (SD)	ANCOVA	LS MD (95 % CI)	Hedges's *g*
**BAI**	32.23 (11.12)	23.77 (10.70)	30.05 (10.75)	26.23 (11.50)	*F*(1;194) = 9.02** η_*p*_^*2*^ = 0.044	3.82 (1.31;6.33)	−0.45
**LSAS**	62.24 (33.11)	47.74 (31.28)	64.15 (35.52)	58.89 (34.49)	*F*(1;192) = 14.66*** η_*p*_^*2*^ = 0.071	9.76 (4.73;14;79)	−0.50
**DASS-21**	33.78 (12.08)	25.55 (12.32)	31.45 (11.75)	28.26 (13.38)	*F*(1;188) = 10.50** η_*p*_^*2*^ = 0.053	4.23 (1.73;7.12)	−0.51
**RSES**	15.11 (5.83)	16.28 (6.36)	15.87 (6.33)	15.79 (6.74)	*F*(1;187) = 4.69* η_*p*_^*2*^ = 0.024	−1.12 (−2.14;–0.10)	0.33
**WHO-QOL**	36.06 (19.85)	44.41 (21.86)	40.54 (19.38)	43.87 (22.64)	*F*(1;187) = 2.87 *η*_*p*_^*2*^ = 0.015	−4.14 (−9.00;0.68)	0.31

*Note*. *p < .05; **p < .01; ***p < .001; IG: Intervention Group; WCG: Waitlist Control Group; BAI: Beck's Anxiety Inventory; LSAS: Liebowitz Social Anxiety Scale; DASS-21: Depression Anxiety Stress Scale; RSES: Rosenberg Self-Esteem Scale; WHOQOL: World Health Organization Quality of Life Brief Questionnaire.

In addition to the ANCOVAs with the CC sample, the RCI for the primary endpoint (BAI score T0–T1) was calculated. The frequencies of improved, unchanged, and deteriorated participants according to the RCI are shown in Table 4. The chi-squared test comparing improved versus not improved participants showed significantly more participants who had improved in the intervention group compared to the control group (χ^2^(1) = 7.75, *p* < .01).

**Table 4:** Reliable Change Index in the control and intervention groups.

RCI	Control *n* (%)	Intervention *n* (%)
Improved	21 (18.9)	38 (36.8)
Unchanged	81 (73.0)	64 (62.1)
Deteriorated	9 (0.1)	3 (0.01)

*Note.* RCI = Reliable Change Index; Improved: χ^2^(1) = 7.75, *p* < .01.

#### 3.3.3 Moderation and subgroup analyses

None of the moderator variables analyzed in this study, including attitude toward online interventions (β = 0.48, SE = 0.27, *p* = .078), treatment expectations (β = 0.88, SE = 1.21, *p* = .47), and the presence of comorbid disorders (β = 0.89, SE = 1.16, *p* = .44), exerted a significant influence on the treatment effect. For detailed results of the moderation analyses, see Supplements, Table G.

The exploratory subgroup analyses for the primary endpoint (change in BAI T0–T1) were conducted for the following groups: age (< 65 years: *n* = 305; ≥ 65 years: *n* = 13), gender (male: *n* = 63; female: *n* = 253), care context (with ongoing psychotherapy: *n* = 132; without psychotherapy: *n* = 186) and medication (with medication: *n* = 129; without medication: *n* = 189) as well as for specific diagnoses (AP: *n* = 111; PD: *n* = 126; SAD: *n* = 56; SP: *n* = 25), analogous to the primary analysis.

**Fig. 2:**
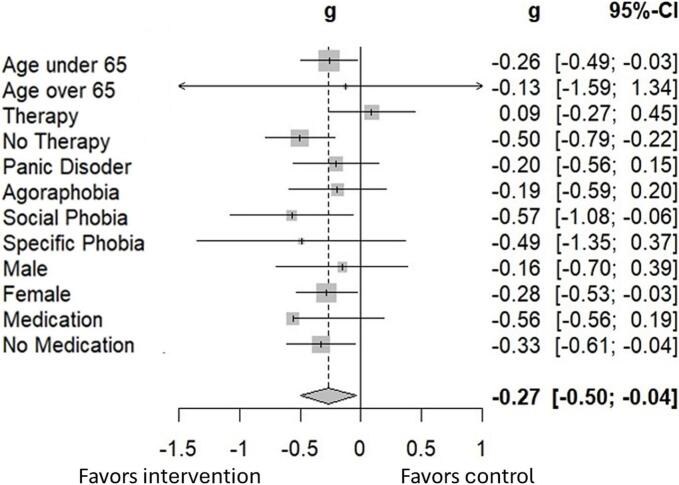
Standardized effect sizes (Hedes's *g*) with 95 % CI of the intention-to-treat ANCOVAs in the subgroups and overall effect across the subgroups.

In the majority of the subgroups, no effect modification by the subgroup variables was observed. However, a notable exception was observed in the subgroup with psychotherapy (“Therapy”). The Hedges's *g* of the respective ANCOVAs are illustrated in Fig. 2. Detailed results of the subgroup analyses can be found in the Supplements, Table H.

The interaction tests of the subgroup variables demonstrate a significant interaction for the care context for the BAI difference value at T1 (*p* < .01). The variables of age, gender, and indication (initial diagnosis) show no significant interactions (see Table 5).

**Table 5:** Interaction test of the subgroup variables in the ITT population (J2R) for the BAI.

	Interaction	Statistics	*p*
*T1*			
	Group x Age	*F*(1; 16,689.4) = 0.19	0.67
	Group x Gender	*F*(1; 5417.2) = 0.37	0.54
	Group x Indication	*F*(4; 4743.9) = 0.35	0.85
	Group x Context of care	*F*(1; 2759.6) = 6.76	< 0.01

*Note*. ITT: intention-to-treat; J2R: jump-to-reference; BAI: Beck's Anxiety Inventory.

## 4 Discussion

In this two-arm randomized controlled trial, we examined the treatment effect of a transdiagnostic internet-based cognitive behavioral therapy intervention (iCBT) for agoraphobia (AP), panic disorder (PD), social anxiety disorder (SAD), and specific phobia (SP) over a period of six weeks (post; T1) with an additional 12-week follow-up (T2).

Participants in the intervention group showed significantly greater improvement in anxiety, depression, and stress, with small-to-medium effect sizes (Hedges's *g* = 0.27–0.35), in the ITT analyses. This effect even withstood a very conservative imputation method (J2R), which can be understood as a modulation of a worst-case scenario (Kenward, 2015). This result is particularly remarkable in a trial with a relatively high dropout rate and speaks for the robustness of the effect. However, the intervention did not increase self-esteem or quality of life immediately after the intervention period. Sensitivity analyses confirmed the results. Yet, at follow-up (12 weeks after baseline), the IG showed significantly greater improvement in self-esteem compared to the WCG, indicating that an increase in self-esteem might be mediated through an improvement in anxiety symptoms in the sense of an indirect and therefore prolonged treatment effect (i.e., the “sleeper effect”). Another possible explanation could be the implementation of the intervention itself in terms of ownership over one's own mental health and the feeling of self-efficacy that is known to be well connected to the concept of self-esteem (Bandura, 1977). The effect sizes found for symptom reduction are smaller compared to the effects found in the meta-analysis by Pauley et al. (2023). However, as stated before, only two of the included studies evaluated comparable (transdiagnostic, unguided) interventions (Bell et al., 2012; Berger et al., 2017). Bell et al. (2012) reported no significant effects on generic anxiety symptoms. Compared to Berger et al. (2017), effect sizes in this study were smaller (BAI, Cohen's *d* = 0.41 post intervention), but the intervention period was longer (nine weeks), which could have contributed to this discrepancy. Guided interventions usually show overall greater effect sizes than unguided programs (see Pauley et al., 2023), which must be noted when interpreting the results of this study. Nevertheless, unguided interventions have several structural and procedural advantages over guided interventions, such as lower costs and easier implementation.

For quality of life, no treatment effect was found. This could be explained by the general assessment of quality of life as a global concept across all domains of the WHOQOL-BREF questionnaire. Some items might not be suitable for adequately capturing the effects of the intervention (e.g., “Do you have enough money to meet your needs?”). Using a disorder-specific scale for health-related quality of life might have been more appropriate.

Subgroup analyses for the primary diagnosis of the participants showed no alteration of the treatment effect, indicating that the transdiagnostic intervention is indeed suitable for various anxiety disorders. In addition, no significant moderation effect of the initial diagnosis (the indication) was found. This is especially noteworthy as the intervention is also indicated for specific phobia. Although anxiety disorders and depression are the disorders targeted most often by iCBT programs, interventions for specific phobias are few. Nevertheless, individuals suffering from specific phobia are less likely to seek professional help and more likely to choose to live with their fears compared to patients with other anxiety disorders, despite severe impairment to their quality of life (Nebel-Schwalm et al., 2022). As the most prevalent anxiety disorder, with a lifetime prevalence of 8.3–13.8 % (Bandelow and Michaelis, 2015), specific phobia in particular needs low-threshold and available treatment options. Therefore, iCBT might be especially useful for patients with specific phobia by lowering barriers to help-seeking behavior such as fear of stigmatization and poor access to professional help (i.e., psychotherapy).

Current treatment situation (with vs. without current psychotherapy), however, yielded modifications of the treatment effect. This was confirmed by the interaction test between the subgroup variable and the primary endpoint (*p* < .01). Concurrent psychotherapy seemed to decrease the effect of the intervention. However, certain considerations must be taken into account when interpreting this result. The intervention was not incorporated in the psychotherapy of the participants in this study, as it would be in a blended-care context; the use of the intervention and of conventional face-to-face psychotherapy in the subgroup with concurrent treatment were independent of each other. This could have been critical if program and therapy addressed different topics simultaneously, for example by providing the patient with different explanatory approaches or methods. It might be better to blend the iCBT program into ongoing psychotherapy, as this has been proven to be effective (Mathiasen et al., 2022).

We assessed attitude toward online interventions as a further potential moderator. However, in contrast to the findings reported by Schröder et al. (2017), who examined the impact of the Novego program (then known as ConfID) on anxiety symptoms, attitude toward online interventions did not yield a moderating effect. This discrepancy could be attributed to the growing use of digital technology. At the time of the study conducted by Schröder et al. (2017) in 2014, internet-based interventions were less common than now, and people may have been generally more reluctant or skeptical about the effectiveness and benefits of such programs. This suggests that individual attitudes toward the programs may have had a greater influence on the success of the therapy. Attitudes toward online interventions were positive on average in this study, with low variance, which explains the failure to detect a moderating effect.

## 5 Limitations

When interpreting these results, some limitations must be considered. First, generalizability might be limited due to the overrepresentation of middle-aged, well-educated females in this all-German sample. Even though the sample is representative of the population of individuals with anxiety diagnoses, the effects might not be transferable without limitations to samples with different characteristics. Also, study and intervention adherence were low. The subgroup and moderation analyses must be understood exploratively as the study was not powered for such analyses. Further, the Novego program has the advantage for the user that the content is available beyond the intended intervention period of 12 months. However, in this study only a follow-up period of six weeks after the post assessment was evaluated. Therefore, no claims about the sustainability of the treatment effects can be made. In addition, it must be considered that the sensitivity analyses, specifically the PP analysis, must be interpreted with caution due to potential confounding variables. We did not adjust for biases due to differential loss to follow-up for the PP analysis and therefore the validity of the effect estimates is limited (Hernán and Robins, 2017). The effect of the PP analysis must be seen as an estimate under optimal circumstances and therefore cannot be regarded as the actual treatment effect.

Future studies might focus on fully powered, stratified trials regarding particular patient characteristics such as primary diagnosis and therapy experience. As adherence in this study was low, investigating reasons for dropout and treatment discontinuation might help further improve this and other iCBT programs, especially since adherence is a known problem in trials on internet-based interventions (Karyotaki et al., 2015). More effort should be made to determine what is effective for whom and the potential underlying mechanisms of the efficacy. Moreover, as adherence is a common problem but iCBT is still found to be generally effective in RCTs, investigating the influence of “dosage,” defining this term, and providing an evidence-based optimal dosage for iCBT seems necessary.

## 6 Conclusion

This study demonstrates the effectiveness of a transdiagnostic internet-based cognitive behavioral therapy (iCBT) intervention in reducing anxiety symptoms across various anxiety disorders, including agoraphobia, panic disorder, social anxiety disorder, and specific phobia. The findings suggest that while the intervention positively influences anxiety and related symptoms, its immediate impact on self-esteem and quality of life is limited, although a late-onset effect on self-esteem was observed at follow-up. The results underscore the potential of iCBT as a low-threshold treatment option, particularly for specific phobias, which are often underrepresented in iCBT research. However, conservative ITT analysis yielded only small effect sizes, which must be taken into account when interpreting the clinical relevance of the results. Additionally, the presence of concurrent psychotherapy may reduce the treatment effect of the iCBT intervention, indicating the need for future research to explore integrated therapeutic approaches. Despite its limitations, in our opinion this study contributes valuable insights into the role of iCBT in addressing anxiety disorders. Further research is needed to better understand the mechanisms underlying treatment efficacy, such as the role of adherence.

## Declaration of Generative AI and AI-assisted technologies in the writing process

During the preparation of this work the authors used the institutional access through University of Hamburg to OpenAI API (ChatGPT, model GPT 4 omni mini) to improve the clarity and conciseness of the writing. After using this tool, the authors reviewed and edited the content as needed, and they take full responsibility for the content of the published article.

## Funding

This study was supported by IVPNetworks GmbH, Conventstr. 8-10, 22089 Hamburg, Germany.

## CRediT authorship contribution statement

**Anna Baumeister:** Conceptualization, Project administration, Data curation, Formal analysis, Investigation, Writing – original draft. **Lea Schuurmans:** Formal analysis, Investigation, Writing – original draft. **Steffen Moritz:** Conceptualization, Methodology, Writing – review & editing, Supervision.

## Declaration of competing interest

The authors declare the following financial interests/personal relationships which may be considered as potential competing interests: Steffen Moritz reports equipment, drugs, or supplies was provided by IVPNetworks GmbH. If there are other authors, they declare that they have no known competing financial interests or personal relationships that could have appeared to influence the work reported in this paper.

## Appendix A Supplementary data

Supplementary tables Image 1
