# Supplementary material for: Anxiety unplugged: Effectiveness of an unguided, transdiagnostic, web-based intervention for anxiety disorders—A randomized controlled trial
Source: Internet Interv. 2025 Aug 8;41:100867. doi: 10.1016/j.invent.2025.100867 (PMC12362362; doi:10.1016/j.invent.2025.100867)
Supplement: Supplementary file 1 — Supplementary tables [file mmc1.docx]

**SUPPLEMENTS**

**Table A.** Differences between the groups of the intention-to-treat sample at follow-up (ANCOVAs with baseline values and care context as covariates), imputed with jump-to-reference, showing least square mean differences (LS MD) with 95% CI, *p*-value, and effect sizes.

|  | | **95% CI** | |  |  |  |  |
| --- | --- | --- | --- | --- | --- | --- | --- |
|  | **LS MD** | **Lower** | **Upper** | ***F*-Statistics** | ***p*** | **η_p_^2^** | **Hedges’s *g*** |
| **BAI** | 2.47 | 0.14 | 4.80 | *F*(1;2746.211) = 4.35 | .037 | 0.019 | 0.24 |
| **LSAS** | 6.62 | 1.35 | 11.88 | *F*(1;2622.424) = 6.08 | .014 | 0.026 | 0.30 |
| **DASS-21** | 2.53 | –0.06 | 5.13 | *F*(1;3048.662) = 3.68 | .055 | 0.016 | 0.23 |
| **RSES** | –1.13 | –2.20 | –0.07 | *F*(1;1838.941) = 4.34 | .037 | 0.020 | –0.12 |
| **WHOQOL** | –2.27 | –6.71 | 2.16 | *F*(1;4422.53) = 1.07 | .302 | 0.005 | –0.27 |

*Note***.** BAI: Beck’s Anxiety Inventory; LSAS: Liebowitz Social Anxiety Scale; DASS-21: Depression Anxiety Stress Scale; RSES: Rosenberg Self-Esteem Scale; WHOQOL: World Health Organization Quality of Life Brief Questionnaire.

**Table B.** Differences between the groups of the intention-to-treat sample at T1 (ANCOVAs with baseline values and care context as covariates), imputed with multiple imputation by chained equations, showing least square mean differences (LS MD) with 95% CI, p-value and effect size η_p_^2^ and Hedges’s *g*.

|  | | **95%CI** | |  |  |  |  |
| --- | --- | --- | --- | --- | --- | --- | --- |
|  | **LS MD** | **Lower** | **Upper** | ***F*-Statistics** | ***p*** | ***η_p_^2^*** | **Hedges’s *g*** |
| **BAI** | 3.46 | 1.18 | 5.75 | *F*(1;551.01) = 8.81 | .003 | 0.033 | 0.36 |
| **LSAS** | 9.84 | 5.00 | 14.67 | *F*(1;494.84) = 14.44 | .000 | 0.057 | 0.52 |
| **DASS-21** | 4.27 | 1.73 | 6.81 | *F*(1;398.06) = 11.27 | .001 | 0.049 | 0.43 |
| **RSES** | -1.02 | -2.02 | -0.02 | *F*(1;322.05) = 4.03 | .046 | 0.022 | -0.28 |
| **WHOQOL** | -4.29 | -10.02 | 1.44 | *F*(1;395.39) = 1.14 | .140 | 0.017 | -0.24 |

*Note***:** BAI: Beck’s Anxiety Inventory; LSAS: Liebowitz Social Anxiety Scale; DASS-21: Depression Anxiety Stress Scale; RSES: Rosenberg Self-Esteem Scale; WHOQOL: World Health Organization Quality of Life Brief Questionnaire.

**Table C.** Differences between the groups of the complete case sample over time (baseline to post), mean values and standard deviations (*N* = 201).

|  | **IG (*n* = 95)** | | **WCG (*n* = 106)** | | **Differences between the groups from baseline to post** | | |
| --- | --- | --- | --- | --- | --- | --- | --- |
|  | **Baseline *M* (*SD*)** | **Post *M* (*SD*)** | **Baseline *M* (*SD*)** | **Post *M* (SD)** | **ANCOVA** | **LS MD (95% CI)** | **Hedges’s *g*** |
| **BAI** | 31.02 (11.47) | 23.68 (10.94) | 30.05 (10.75) | 26.23 (11.50) | *F*(1;210) = 6.91**  *η_p_^2^* = 0.032 | 3.17 (0.79;5.54) | –0.37 |
| **LSAS** | 61.32 (32.24) | 47.19 (30.73) | 64.15 (35.52) | 58.89 (34.49) | *F*(1;208) =15.47***  *η_p_^2^* = 0.069 | 9.56 (4.77;14.35) | –0.48 |
| **DASS-21** | 33.22 (12.00) | 25.21 (12.34) | 31.45 (11.75) | 28.26(13.38) | *F*(1;202) = 10.48**  *η_p_^2^* = 0.059 | 4.30 (1.68;6.92) | –0.48 |
| **RSES** | 15.50 (5.95) | 16.64 (6.49) | 15.87 (6.33) | 15.79 (6.74) | *F*(1;201) = 5.19*  *η_p_^2^* = 0.025 | –1.11 (–2.07;–0.15) | 0.33 |
| **WHO-QOL** | 37.86 (20.60) | 46.09 (22.56) | 40.54 (19.38) | 43.87 (22.64) | *F*(1;201) = 3.26  *η_p_^2^* = 0.016 | –4.18 (–8.75;0.39) | 0.29 |

*Note*. * *p* < .05; ** *p* < .01; *** *p* < .001; IG: intervention group; WCG: waitlist control group; BAI: Beck’s Anxiety Inventory; LSAS: Liebowitz Social Anxiety Scale; DASS-21: Depression Anxiety Stress Scale; RSES: Rosenberg Self-Esteem Scale; WHOQOL: World Health Organization Quality of Life Brief Questionnaire.

**Table D.** Differences between the groups of the intention-to-treat sample at T2 (ANCOVAs with baseline values and care context as covariates), imputed with multiple imputation by chained equations, showing least square mean differences (LS MD) with 95% CI, *p*-value and effect size η_p_^2^ and Hedges’s *g*.

|  | | **95%CI** | |  |  |  |  |
| --- | --- | --- | --- | --- | --- | --- | --- |
|  | **LS MD** | **Lower** | **Upper** | ***F*-Statistics** | ***p*** | ***η_p_^2^*** | **Hedges’s *g*** |
| **BAI** | 2.78 | 0.27 | 5.29 | *F*(1;400.46) = 4.62 | .030 | 0.017 | 0.25 |
| **LSAS** | 8.58 | 2.57 | 14.60 | *F*(1;396.64) = 5.97 | .005 | 0.025 | 0.35 |
| **DASS-21** | 2.60 | -0.53 | 5.72 | *F*(1;272.75) = 2.96 | .102 | 0.015 | 0.22 |
| **RSES** | -1.56 | -2.70 | -0.41 | *F*(1;381.90) = 7.21 | .008 | 0.033 | -0.36 |
| **WHOQOL** | -2.93 | -7.71 | 1.85 | *F*(1;1200.89) = 1.55 | .228 | 0.006 | -0.14 |

*Note***:** BAI: Beck’s Anxiety Inventory; LSAS: Liebowitz Social Anxiety Scale; DASS-21: Depression Anxiety Stress Scale; RSES: Rosenberg Self-Esteem Scale; WHOQOL: World Health Organization Quality of Life Brief Questionnaire.

**Table E**. Differences between the groups of the complete case sample over time (baseline to follow-up), mean values and standard deviations (*N* = 212).

|  | **IG (*n* = 105)** | | **WCG (*n* = 107)** | | **Differences between the groups from baseline to follow-up** | |  |
| --- | --- | --- | --- | --- | --- | --- | --- |
|  | **Baseline *M* (*SD*)** | **Follow-up *M* (*SD*)** | **Baseline *M* (*SD*)** | **Follow-up *M* (*SD*)** | **ANCOVA** | **LS MD (95% CI)** | **Hedges’s *g*** |
| **BAI** | 30.28 (11.54) | 22.15 (10.44) | 29.49 (10.36) | 25.54 (11.03) | *F*(1;211) = 9.79**  *η_p_^2^* = 0.044 | 3.82 (1.41;6.23) | –0.41 |
| **LSAS** | 61.44 (32.64) | 45.58 (32.04) | 63.64 (35.89) | 57.42 (34.73) | *F*(1;210) = 13.22***  *η_p_^2^* = 0.059 | 10.32 (4.72;15.91) | –0.44 |
| **DASS–21** | 33.46 (12.10) | 26.14 (12.79) | 31.50 (11.77) | 28.76 (13.29) | *F*(1;209) = 7.55**  *η_p_^2^* = 0.035 | 3.93 (1.11;6.75) | –0.41 |
| **RSES** | 15.94 (5.88) | 17.24 (6.51) | 16.07 (6.06) | 15.70 (6.35) | *F*(1;208) = 9.70**  *η_p_^2^* = 0.045 | –1.74 (–2.83;–0.64) | 0.43 |
| **WHO–QOL** | 37.73 (19.78) | 44.76 (21.70) | 39.72 (18.79) | 43.69 (22.48) | *F*(1;208) = 1.07  *η_p_^2^* = 0.005 | –2.56 (–7.45;2.32) | 0.17 |

*Note*. * *p* < .05; ** *p* < .01; *** *p* < .001; IG: intervention group; WCG: waitlist control group; BAI: Beck’s Anxiety Inventory; LSAS: Liebowitz Social Anxiety Scale; DASS-21: Depression Anxiety Stress Scale; RSES: Rosenberg Self–Esteem Scale; WHOQOL: World Health Organization Quality of Life Brief Questionnaire.

**Table F.** Differences between the groups of the per protocol sample over time (baseline to follow-up), mean values and standard deviations (*N* = 191).

|  | **IG (*n* = 85)** | | **WCG (*n* = 106)** | | **Differences between the groups from baseline to follow–up** | | |
| --- | --- | --- | --- | --- | --- | --- | --- |
|  | **Baseline *M* (*SD*)** | **Follow–up *M* (SD)** | **Baseline *M* (*SD*)** | **Follow–up *M* (*SD*)** | **ANCOVA** | **LS MD**  **(95% CI)** | **Hedges’s *g*** |
| **BAI** | 31.04 (11.04) | 21.48 (10.31) | 29.49 (10.36) | 25.54 (11.03) | *F*(1;188) = 14.49***  *η_p_^2^* = 0.072 | 4.94 (2.38;7.49) | –0.56 |
| **LSAS** | 60.56 (33.99) | 43.12 (32.21) | 63.64 (35.89) | 57.42 (34.73) | *F*(1;188) = 15.99***  *η_p_^2^* = 0.078 | 11.96 (6.06;17.86) | –0.51 |
| **DASS–21** | 33.47 (11.95) | 24.85 (12.54) | 31.50 (11.77) | 28.76 (13.29) | *F*(1;187) = 11.41***  *η_p_^2^* = 0.058 | 5.15 (2.14;8.16) | –0.52 |
| **RSES** | 15.75 (5.98) | 17.39 (6.55) | 16.07 (6.06) | 15.70 (6.35) | *F*(1;187) = 10.84**  *η_p_^2^* = 0.055 | –1.93 (–3.08;–0.77) | 0.48 |
| **WHO–QOL** | 35.15 (18.45) | 44.35 (21.20) | 39.72 (18.79) | 43.69 (22.48) | *F*(1;187) = 1.83  *η_p_^2^* = 0.010 | –3.57 (–8.78;1.64) | 0.26 |

*Note*. * *p* < .05; ** *p* < .01; *** *p* < .001; IG: intervention group; WCG: waitlist control group; BAI: Beck’s Anxiety Inventory; LSAS: Liebowitz Social Anxiety Scale; DASS-21: Depression Anxiety Stress Scale; RSES: Rosenberg Self–Esteem Scale; WHOQOL: World Health Organization Quality of Life Brief Questionnaire.

**Table G.** Estimates (SE) of the moderation analyses in the per protocol sample.

|  | **Effect** | **Estimate** | **SE** | ***p*** |
| --- | --- | --- | --- | --- |
| APOI |  |  |  |  |
|  | (Intercept) | 7.79 | 10.05 | .440 |
|  | group | –22.06 | 15.12 | .146 |
|  | APOI | –0.07 | 0.18 | .692 |
|  | group*APOI | 0.48 | 0.27 | .078 |
| TEX-Q |  |  |  |  |
|  | (Intercept) | 1.54 | 4.93 | .755 |
|  | group | –1.24 | 8.25 | .880 |
|  | TEX-Q | 0.34 | 0.72 | .639 |
|  | group*TEX-Q | 0.88 | 1.21 | .471 |
| WSQ |  |  |  |  |
|  | (Intercept) | 5.90 | 2.39 | .015 |
|  | group | 1.96 | 3.79 | .607 |
|  | WSQ | –0.69 | 0.74 | .348 |
|  | group*WSQ | 0.89 | 1.16 | .443 |

*Note*. Outcome variable: BAI T0-T1; APOI: Attitude Towards Psychological Online Interventions; TEX-Q: Treatment Expectation Questionnaire; WSQ: Web Screening Questionnaire (number of comorbid disorders).

**Table H.** Results of ANCOVAs of the subgroup analyses for the primary endpoint (BAI T0-T1) in the ITT sample (imputation of missing values with jump-to-reference method).**
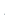
**

|  |  |  | **95% CI** | |  |  |  |
| --- | --- | --- | --- | --- | --- | --- | --- |
| **Subgroup** | ***n*** | **LSMD** | **Lower** | **Upper** | ***F*-Statistics** | ***p*** | **Hedges' *g*** |
| Age Under 65 | 305 | 2.59 | 0.27 | 4.90 | *F*(1; 2127.98) = 4.80 | .029 | -0.26 |
| Age Over 65 | 13 | 1.18 | -16.22 | 18.59 | *F*(1; 8444.59) = 0.25 | .615 | -0.13 |
| Therapy | 132 | -0.85 | -4.35 | 2.65 | *F*(1; 4806.26) = 0.35 | .557 | 0.09 |
| No Therapy | 186 | 5.07 | 2.17 | 7.97 | *F*(1; 2020.48) = 11.49 | .001 | -0.51 |
| Diagnosis: Panic | 126 | 2.11 | -1.59 | 5.82 | *F*(1; 2604.59) = 1.31 | .253 | -0.21 |
| Diagnosis: Agora | 111 | 1.82 | -1.98 | 5.61 | *F*(1; 4345.98) = 0.91 | .341 | -0.19 |
| Diagnosis: Social | 56 | 5.40 | 0.47 | 10.33 | *F*(1; 2686.51) = 4.77 | .029 | -0.57 |
| Diagnosis: Specific | 25 | 4.92 | -4.38 | 14.21 | *F*(1; 5641.10) = 1.25 | .264 | -0.49 |
| Male | 63 | 1.40 | -3.61 | 6.42 | *F*(1; 3142.30) = 0.44 | .509 | -0.16 |
| Female | 253 | 2.83 | 0.29 | 5.37 | *F*(1; 2767.82) = 4.80 | .028 | -0.28 |
| Medication | 129 | 1.80 | -1.85 | 5.45 | *F*(1; 2367.09) = 1.00 | .317 | -0.19 |
| No Medication | 189 | 3.27 | 0.41 | 6.13 | *F*(1; 2974.91) = 5.06 | .025 | -0.33 |

*Note.* LSMD: Least Square Mean Difference; CI: Confidence interval.
